# Obstetrics, Gynecology and Pelvic Surgery

**Published:** 1896-06

**Authors:** Electa B. Whipple


### OBSTETRICS, GYNECOLOGY AND PELVIC SURGERY.

CONDUCTED BY ELECTA B. WHIPPLE, A. M., M. D.

### VAGINAL FIXATION OF THE UTERUS.

WERTHEIM, of Vienna, (*Centralblatt für Gynäkologie*, May 4, 1895, quoted in *British Gynecological Journal*,) reports results of thirty-seven vaginal fixations of the uterus in the clinic of Professor Schauta. In seven cases operated upon after the directions of Mackenrodt, return of the diseased condition took place. Of nine cases operated upon by Dührssen's (without passing the sound into the uterus) six were completely cured and in three a relapse occurred. Ten cases operated upon by the open method of Dührssen (with the opening of the plica vesica uterina) were cured. Five cases were operated upon by the recent modification of Mackenrodt; four of these were completely cured and in one accidental prolapse of the bowels occurred. Of the six remaining cases, also operated upon by Dührssen's open method—of recent duration—it was impossible to judge of final results. Wertheim concludes that the peritoneum should always be opened in operating and only with this stipulation is the method of Dührssen fully secured.

Mackenrodt's method consists in seizing the cervix with volsella and drawing it firmly backward. A long incision is made in the anterior wall up to the cervix. The bladder is pushed upward and the anterior uterine wall, by successive sutures in the vaginal incision, is caught as high up as possible and at the same time fixed to the incision of the vaginal wall. By Dührssen's method the fundus of the uterus is drawn into the incision, after which it is easy to draw down and open the peritoneum, and by his open method the versico uterine fold of the peritoneum is opened and then the fundus of the uterus can be fixed in the vaginal incision without any force.

---

### SUGGESTIONS FROM PRACTICAL OBSERVATION IN OBSTETRICS.

STAPLES (*Annals of Gynecology and Pediatrics*, December, 1895; *American Gynecological and Obstetrical Journal*) says that of 1,000 recorded cases 33 per cent. were primaparæ. The largest number of births was in the months of August and October, the smallest in June. The youngest mother was 14 years 3 months; child weighed five and one-half pounds; full term. Vertex presentations, 90 per cent.; face, 4 per cent.; podalic, 5 per cent. In cases of dystocia, he cautions against too hasty interference before full dilatation of the cervix and the neglect of anodynes and rest, and adopts Goodell's rule, that in uniformly contracted pelves the use of forceps is better than version. In narrowing of the conjugata vera, version should be resorted to after a trial with forceps, version being preferable to lashing of the forceps' handles. Injuries during labor will occur even with the utmost care. Those who claim never to have seen a laceration may be suspected of being very careless observers or unfortunate in their early moral education. Lacerations of both cervix and perineum should be repaired at once under an anesthetic and with aseptic precautions. He urges the importance of a careful study of each case before delivery as to pelvic diameters, the general condition of patient, especially nephritic disturbances, and urges thorough antiseptic precautions both during delivery and the puerperal state.

---

### BLEEDING FROM VARICOSE VEINS OF THE VAGINA AND VULVA AS A COMPLICATION OF LABOR.

THIELE (*Deutsche Medicinische Wochenschrift; American Journal of Medical Sciences*) reports the case of a multipara who was seized with hemorrhage from the vagina. On examination the source of the bleeding was found to be varicose veins in that region. The hemorrhage, which became severe, was checked with applications of ice and iodoform-gauze tampon. Repeated transfusion of saline solutions was also made. Labor began and the patient was delivered spontaneously of a dead child. Profuse hemorrhage from the vagina and urethra occurred. Patient made a slow but uninterrupted recovery.

---

### USE OF THE PELVIMETER.

Dr. Austin Flint advocates use of pelvimeter for every primipara, (*New York Medical Record; American Journal of Surgery and Gynecology,*) and says that in pelves with a true conjugate diameter of less than three and one-half inches operative interference usually is imperative. Just under three and one-half inches either forceps or version may be employed. Pelves in which the true conjugate is much less than three and one-half inches call for more severe operations, notab ly the Cesarean section.

---

### THE TREATMENT OF DYSMENORRHEA.

Dr. J. C. Chapman (*Alabama Medical and Surgical Age*) has found apiol, viburnum prunifolium and dioviburnia useful agents in the treatment of dysmenorrhea.

---

### FEMALE PELVIS IN PRIMITIVE RACES.

Straty (*Nederlandsch Tifdschrift voor Verloskunde en Gynäkologie; Med. Age*) investigated a series of cases in Java to test the accuracy of certain theories in respect to the relative characters of the pelvis in European and in barbarian or semi-civilised women. Faayer, of Leyden, declared twenty years ago that the Javanese pelvis was unusually round at the inlet. Stratz reminds obstetricians that this theory was based on the examination of a few macerated pelves. He therefore measured a large number of pelves of Javanese women living up-country. Two races were included in his series, the more primitive being darker, more slender and smaller. The measurements showed little or no difference more than could be explained by the small general proportions of one of the races. The same may be said of the difference between the average Javanese and European pelvis. As it happens, however, the theory of

Faayer seems substantially correct, the transverse measurement of the Javanese pelvis being on an average relatively small. The obstetric teacher should also bear in mind that Stratz found plenty of contracted pelves among these primitive women, who escaping the evils of civilisation do not enjoy its benefits.

---

### TO RELIEVE BLADDER SYMPTOMS.

Dr. Baldy (*Journal of Practical Medicine*) has found that bladder symptoms complicate a supposed pelvic trouble in women and that painful and frequent micturition, irritability of the bladder, bearing-down pains and bladder distress are relieved in a considerable number of patients by simple dilatation of the urethra. Even in case of true cystitis, urethral dilatation, accompanied by alkaline diuretics and bladder irrigation, is invaluable.

---

